# Supplementary material for: The Effectiveness of Clinician-Led Community-Based Group Exercise Interventions on Health Outcomes in Adults with Type 2 Diabetes Mellitus: A Systematic Review and Meta-Analysis
Source: Int J Environ Res Public Health. 2024 May 7;21(5):601. doi: 10.3390/ijerph21050601 (PMC11120654; doi:10.3390/ijerph21050601)
Supplement: Supplementary file 1 [file ijerph-21-00601-s001.zip › ijerph-2945000-supplementary.pdf]

SUPPLEMENTARY FILE – MeSH Terms and Database Searches

Table S1. MeSH Terms and Database Searches

Database	MeSH Terms and Search Strategy
**Medline via Ovid**	1. exp Diabetes Mellitus, Type 2/<br>2. (Type* adj3 ("2" or "II" or two*) adj3 (diabete* or diabetic*)).ti,ab.<br>3. ((Maturit* or adult* or slow*) adj3 onset* adj3 (diabete* or diabetic*)).ti,ab.<br>4. ((Ketosis-resistant* or stable*) adj3 (diabete* or diabetic*)).ti,ab.<br>5. (((Non-insulin* or Non insulin* or Noninsulin*) adj3 depend* adj3 (diabete* or diabetic*)) or (NIDDM or T2DM or T2D)).ti,ab.<br>6. or/1-5<br>7. exp Exercise Therapy/<br>8. Exercise/<br>9. Physical Fitness/<br>10. (physical activit* or exercise or fitness or group exercise or strength training).ti,ab.<br>11. (group adj2 (exercise or fitness)).ti,ab.<br>12. (functional adj2 (fitness or training)).ti,ab.<br>13. or/7-12<br>14. exp Health Personnel/<br>15. (clinician* or physiotherap* or physical therap* or exercise physiologist* or personal trainer* or trainer* or health educator* or allied health personnel or lead* or led or supervis*).mp.<br>16. or/14-15<br>17. Community Health Centers/<br>18. (communit* or community based or community centre* or community center* or fitness centre* or fitness center* or gym* or community hall*).mp.<br>19. or/17-18<br>20. and/6,13,16,19<br>21. adult/ or exp aged/ or middle aged/ or young adult/<br>22. (adult* or young adult* or middle-aged or elder* or older adult*).mp.<br>23. or/21-22<br>24. and/20,23<br>25. limit 24 to english language<br>26. limit 25 to yr="2003 -2023"
**Embase via Ovid**	1. non insulin dependent diabetes mellitus/<br>2. (Type* adj3 ("2" or "II" or two*) adj3 (diabete* or diabetic*)).ti,ab.<br>3. ((Maturit* or adult* or slow*) adj3 onset* adj3 (diabete* or diabetic*)).ti,ab.<br>4. ((Ketosis-resistant* or stable*) adj3 (diabete* or diabetic*)).ti,ab.<br>5. (((Non-insulin* or Non insulin* or Noninsulin*) adj3 depend* adj3 (diabete* or diabetic*)) or (NIDDM or T2DM or T2D)).ti,ab.<br>6. or/1-5<br>7. exp kinesiotherapy/<br>8. exp Exercise/<br>9. Fitness/<br>10. (physical activit* or exercise or fitness or group exercise or strength training).ti,ab.<br>11. (group adj2 (exercise or fitness)).ti,ab.<br>12. (functional adj2 (fitness or training)).ti,ab.<br>13. or/7-12<br>14. exp Health care Personnel/<br>15. (clinician* or physiotherap* or physical therap* or exercise physiologist* or personal trainer* or trainer* or health educator* or allied health personnel or lead* or led or supervis*).mp.<br>16. or/14-15<br>17. health center/

	18. (communit* or community based or community centre* or community center* or fitness centre* or fitness center* or gym* or community hall*).mp.<br>19. or/17-18<br>20. and/6,13,16,19<br>21. adult/ or exp aged/ or middle aged/ or young adult/<br>22. (adult* or young adult* or middle-aged or elder* or older adult*).mp.<br>23. or/21-22<br>24. and/20,23<br>25. limit 24 to english language<br>26. limit 25 to yr="2003 -2023"<br>27. limit 26 to "remove medline records"<br>28. limit 27 to conference abstract<br>29. 27 not 28
**Scopus**	((TITLE-ABS-KEY (((type* W/3 ("2" OR "II" OR two*) W/3 (diabete* OR diabetic*)))) OR TITLE-ABS-KEY (((maturit* OR adult* OR slow*) W/3 onset* W/3 (diabete* OR diabetic*))) OR TITLE-ABS-KEY ((("Ketosis-resistant*" OR stable*) W/3 (diabete* OR diabetic*))) OR TITLE-ABS-KEY ((("Non-insulin*" OR "Non insulin*" OR noninsulin*) W/3 depend* W/3 (diabete* OR diabetic*)) OR (niddm OR t2dm OR t2d)))) AND (TITLE-ABS-KEY (adult* OR "young adult*" OR "middle-aged" OR elder* OR "older adult*")) AND (TITLE-ABS-KEY (communit* OR "community based" OR "community centre*" OR "community center*" OR "fitness centre*" OR "fitness center*" OR gym* OR "community hall*")) AND (TITLE-ABS-KEY (clinician* OR physiotherap* OR "physical therap*" OR "exercise physiologist*" OR "personal trainer*" OR trainer* OR "health educator*" OR "allied health personnel" OR lead* OR led OR supervis*)) AND ((TITLE-ABS-KEY (("physical activit*" OR "physical fitness" OR "exercise therap*" OR "physical therap*" OR exercise OR fitness OR "group exercise" OR "strength training")) OR TITLE-ABS-KEY ((group) W/2 (exercise OR fitness)) OR TITLE-ABS-KEY ((functional) W/2 (fitness OR training))))) AND PUBYEAR > 2002 AND PUBYEAR < 2023) AND (LIMIT-TO (LANGUAGE , "English"))
**Cinahl Complete via Ebscohost**	S1 (MH "Diabetes Mellitus, Type 2")<br>S2 TI ((Type* N3 ("2" or "II" or two*) N3 (diabete* or diabetic*))) OR AB ((Type* N3 ("2" or "II" or two*) N3 (diabete* or diabetic*)))<br>S3 TI (((Maturit* or adult* or slow*) N3 onset* N3 (diabete* or diabetic*))) OR AB (((Maturit* or adult* or slow*) N3 onset* N3 (diabete* or diabetic*)))<br>S4 TI ((("Ketosis-resistant*" or stable*) N3 (diabete* or diabetic*))) OR AB ((("Ketosis-resistant*" or stable*) N3 (diabete* or diabetic*)))<br>S5 TI ((("Non-insulin*" or "Non insulin*" or Noninsulin*) N3 depend* N3 (diabete* or diabetic*)) or (NIDDM or T2DM or T2D))) OR AB ((("Non-insulin*" or "Non insulin*" or Noninsulin*) N3 depend* N3 (diabete* or diabetic*)) or (NIDDM or T2DM or T2D)))<br>S6 S1 OR S2 OR S3 OR S4 OR S5<br>S7 (MH "Physical Therapy+")<br>S8 (MH "Exercise+")<br>S9 (MH "Physical Fitness+")<br>S10 TI (("physical activit*" or exercise or fitness or "group exercise" or "strength training")) OR AB (("physical activit*" or exercise or fitness or "group exercise" or "strength training"))<br>S11 TI ((group N2 (exercise or fitness))) OR AB ((group N2 (exercise or fitness)))<br>S12 TI ((functional N2 (fitness or training))) OR AB ((functional N2 (fitness or training))<br>S13 S7 OR S8 OR S9 OR S10 OR S11 OR S12<br>S14 (MH "Health Personnel+")<br>S15 TI((clinician* or physiotherap* or "physical therap*" or "exercise physiologist*" or "personal trainer*" or trainer* or "health educator*" or "allied health personnel" or lead* or led or supervis*)) OR AB ((clinician* or physiotherap* or "physical therap*" or "exercise

	physiologist*" or "personal trainer*" or trainer* or "health educator*" or "allied health personnel" or lead* or led or supervis*))<br>S16 S14 OR S15<br>S17 (MH "Community Health Centers+")<br>S18 TI ((communit* or "community based" or "community centre*" or "community center*" or "fitness centre*" or "fitness center*" or gym* or "community hall*") OR ((communit* or "community based" or "community centre*" or "community center*" or "fitness centre*" or "fitness center*" or gym* or "community hall*")<br>S19 S17 OR S18<br>S20 S6 AND S13 AND S16 AND S19<br>S21 (MH "Adult") OR (MH "Aged+") OR (MH "Middle Age") OR (MH "Young Adult")<br>S22 TI ((adult* or "young adult*" or "middle-aged" or elder* or "older adult*")) OR AB ((adult* or "young adult*" or "middle-aged" or elder* or "older adult*"))<br>S23 S21 OR S22<br>S24 S20 AND S23<br>S25 S20 AND S23 Limiters - Publication Date: 20040101-20231231
**PubMed**	(((((("Diabetes Mellitus, Type 2"[Mesh]) OR (NIDDM[Title/Abstract] OR T2DM[Title/Abstract] OR T2D[Title/Abstract])) OR ("type 2 diabetes"[Title/Abstract] OR "adult onset diabetes"[Title/Abstract] OR "slow onset diabetes"[Title/Abstract] OR "ketosis resistant diabetes" OR"non insulin dependent diabetes"[Title/Abstract])) AND (((("Exercise Therapy"[Mesh]) OR "Exercise"[Mesh]) OR "Physical Fitness"[Mesh]) OR ("physical activity"[Title/Abstract] OR exercise[Title/Abstract] OR fitness[Title/Abstract] OR "group exercise"[Title/Abstract] OR "strength training"[Title/Abstract] OR "group fitness"[Title/Abstract] OR "functional fitness"[Title/Abstract] OR "functional training"[Title/Abstract]))) AND (("Health Personnel"[Mesh]) OR (clinician*[Title/Abstract] OR physiotherap*[Title/Abstract] OR "physical therapist"[Title/Abstract] OR "exercise physiologist"[Title/Abstract] OR "personal trainer"[Title/Abstract] OR trainer*[Title/Abstract] OR "health educator"[Title/Abstract] OR "allied health personnel"[Title/Abstract] OR lead*[Title/Abstract] OR led[Title/Abstract] OR supervis*[Title/Abstract]))) AND (("Community Health Centers"[Mesh]) OR (communit*[Title/Abstract] OR "community based"[Title/Abstract] OR "community centre"[Title/Abstract] OR "community centers"[Title/Abstract] OR "fitness centres"[Title/Abstract] OR "fitness centers"[Title/Abstract] OR gyms[Title/Abstract] OR "community halls"[Title/Abstract]))) AND (("Adult"[Mesh]) OR (adult*[Title/Abstract] OR "young adult"[Title/Abstract] OR "young adults"[Title/Abstract] or "middle-aged"[Title/Abstract] OR elder*[Title/Abstract] OR "older adult" OR"older adults"[Title/Abstract]))– limited 2003 – 2023 and English language.
